# A Danish version of the oral health impact profile-14 (OHIP-14): translation and cross-cultural adaptation

**DOI:** 10.1186/s12903-020-01242-z

**Published:** 2020-09-10

**Authors:** Arwa Gera, Paolo M. Cattaneo, Marie A. Cornelis

**Affiliations:** Section of Orthodontics, Department of Dentistry and Oral Health, Aarhus University, Vennelyst Boulevard 9, DK-8000 Aarhus C, Denmark

**Keywords:** Orthodontics, OHIP-14, Oral health-related quality of life, Translation, Cross-cultural adaptation, Danish

## Abstract

**Background:**

The Oral Health Impact Profile-14 (OHIP-14) questionnaire assesses quality of life related to people’s perception of oral disorders on their well-being. However, a translated and validated Danish version of OHIP-14 is not yet available.

The purpose of this pilot study was to translate and cross-culturally adapt the English version of the OHIP-14 into Danish (OHIP-14-DK). In addition, to assess its content and face validity, internal consistency and test-retest reliability.

**Methods:**

The English version of OHIP-14 was translated into Danish following a standard protocol of cross-cultural adaptation. Stages I-IV: translation phase to generate a pre-final version “OHIP-14-DK”. Stage V: pre-testing phase. A random sample of 22 orthodontic patients (mean age 24.7 years, SD ±14.8; 14 females, 8 males) were selected at the Section of Orthodontics, Aarhus University, Denmark. All patients self-completed the OHIP-14-DK and were then interviewed to assess its content and face validity. Internal consistency was assessed with Cronbach’s alpha coefficients. All patients completed the same questionnaire again at a one-week interval. Test-retest reliability was assessed using Spearman’s correlation coefficient and intra-class correlation coefficient (ICC).

**Results:**

The initial and back translations were very similar: the OHIP-14-DK proved to have a good level of equivalence with no translation errors or deviations. Furthermore, the OHIP-14-DK seemed well-adapted to Danish culture and was understood by individuals down to 12 years of age. Pre-testing demonstrated good face and content validity; interviews had a response rate of 100% and confirmed that each item was understandable without inducing reluctance or hesitation. Thus, responses were related to their corresponding item. Therefore, no final adjustments were required for the pre-tested version. Cronbach’s alpha for the OHIP-14-DK subscales fell in the 0.75–0.84 range, indicating an adequate-to-good internal consistency. Spearman’s correlation coefficient for the OHIP-14-DK total score was 0.77. The ICC for the OHIP-14-DK total score was 0.91.

**Conclusions:**

The OHIP-14-DK seems well adapted to Danish culture, proved to be face and content valid and also showed good internal consistency and excellent reliability. However, its psychometric properties still need to be tested.

**Study registration:**

Not applicable

## Background

Malocclusion, as an oral deviation from the norm, is highly prevalent and can unfavourably affect patients’ social, physical and psychological well-being [1–3]. Hence, it is crucial to take into account patients’ oral health-related quality of life (OHRQoL) when performing orthodontic treatments.

In 1995, The World Health Organization (WHO) recognized the importance of evaluating and improving people’s quality of life (QoL) [4]. The WHO defined QoL as “An individual’s perception of their position in life in the context of culture and value systems in which they live and in relation to their goals, expectations, standards and concerns. It is a broad-ranging concept affected in a complex way by the person’s physical health, psychological state, personal beliefs, social relationships and their relationships to salient features of their environment” [5]. However, there is a conceptual and methodological debate about the meaning of QoL and about what should be measured. The concept of QoL has no uniform definition [6, 7].

Health as related to QoL was first mentioned in the medical literature in the middle of the twentieth century [8]. The WHO (1946) adopted the definition of health as “A state of complete physical, mental and social well-being and not merely the absence of disease or infirmity” [9]. This marked a milestone in associating health with QoL, giving rise to the term health-related quality of life (HRQoL), making the use of health status measures fundamental. HRQoL is also defined as “A term referring to the health aspects of quality of life, generally considered to reflect the impact of disease and treatment on disability and daily functioning; it has also been considered to reflect the impact of perceived health on an individual’s ability to live a fulfilling life. However, more specifically, HRQoL is a measure of the value assigned to duration of life as modified by impairments, functional states, perceptions and opportunities, as influenced by disease, injury, treatment and policy [4]. Guyatt et al. defined HRQoL as “a multi-dimensional concept that is related to physical, psychological, emotional, and social functioning and hence representing the overall health of an individual, going beyond direct measures of population health, life expectancy, and causes of death, and focusing on the impact that health status has on QoL”. The same authors emphasize that “a related concept of HRQoL is well-being, which assesses the positive aspects of a person’s life, such as positive emotions and life satisfaction” [10].

Whereas the use of HRQoL measures is well established in the medical field, their use in dentistry is still not widespread [11]. In dentistry, objective measures of oral disease or malocclusion reflect only the endpoint of the disorder or the malocclusion process; they do not reflect any insight into the impact that oral disorders have on the individuals’ orofacial function, psychosocial well-being or QoL. The need to develop patient-based measures of oral health status was first recognized by Cohen and Jago in 1976 [12], who indicated a lack of data related to the psychosocial impact of oral health problems at the time. In addition, Reisine et al. (1989) found that orofacial function and health were important aspects of an individual’s general health and QoL [13].

OHRQoL is a concept that includes subjective evaluation of perceived physical, psychological and social aspects of oral health. The use of OHRQoL measures has important implications for research, public health and clinical practice. Among others, health measure outcomes help promote health, evaluate the effectiveness and efficiency of healthcare systems, and serve as a medical audit [11].

Until two decades ago, indices to measure OHRQoL were virtually absent. Currently, an impressive range of OHRQoL instruments exists [14]. Nevertheless, methodological developments in this area are still ongoing. Moreover, when a questionnaire is to be used in a different setting than the original measure, a process of adaptation for its use in a different setting is needed, which is recognized as “cross-cultural adaptation of self-reported measures” [15]. Cross-cultural adaptation is the process of preparing a questionnaire for use in another setting, starting by looking into language (translation) and cultural adaptation issues, aiming to reach equivalence between the original source and the target versions of the questionnaire. Besides the linguistic translation part, all items must also be culturally adapted to maintain the content validity of the instrument across different cultures. Therefore, cross-cultural adaptation consists of two components: translation and adaptation to idiom, cultural context, and lifestyle of the target culture.

The Oral Health Impact Profile-49 (OHIP-49), described by Slade and Spencer in English language in Australia, is a self-reported OHRQoL questionnaire designed to assess QoL related to people’s perception of the impact of oral disorders on their well-being. It is widely used [16, 17] and consists of 49 items that are divided into seven domains (functional limitation, physical pain, psychological discomfort, physical disability, psychological disability, social disability and handicap) [18]. However, due to the extensiveness of the OHIP-49, a shortened version (OHIP-14) consisting of only 14 items was developed while retaining the original conceptual dimensions (see Appendix A.1.). It is a useful tool in clinical settings [19], and is reliable and valid [18, 20]. In fact, the OHIP-14 is the most widely available OHRQoL instrument, recording almost 1000 citations in Scopus. However, despite its widespread use, Denmark lacks a translated and cross-culturally adapted Danish version of the OHIP-14.

Health status measures or self-satisfaction measures need to be adapted for use in multinational and multicultural research. The OHIP has been translated into 24 languages [21]. Several studies used OHIP-14 in Denmark. The majority [22–24] used the original English OHIP-14, whereas one study [25] reported using a translated Danish version (with no reference provided) and another study [26] reported the translation of the OHIP-49 into Danish, but without following the guidelines for translation and cross-cultural adaptation of questionnaires [27]. In the literature, there is neither a published nor a validated Danish version.

The purpose of this pilot study was to translate and cross-culturally adapt the original English version of OHIP-14 into Danish (OHIP-14-DK). In addition, to assess its content and face validity, internal consistency and test re-test reliability.

## Methods

This study was conducted between September and October 2018 in Denmark at the Section of Orthodontics, Department of Dentistry and Oral Health, Aarhus University. This type of study is exempt from ethical approval in Denmark (Scientific Ethical Committee for Central Jutland, Denmark, case no. 1–10–72-148-19).

**Fig. 1:**
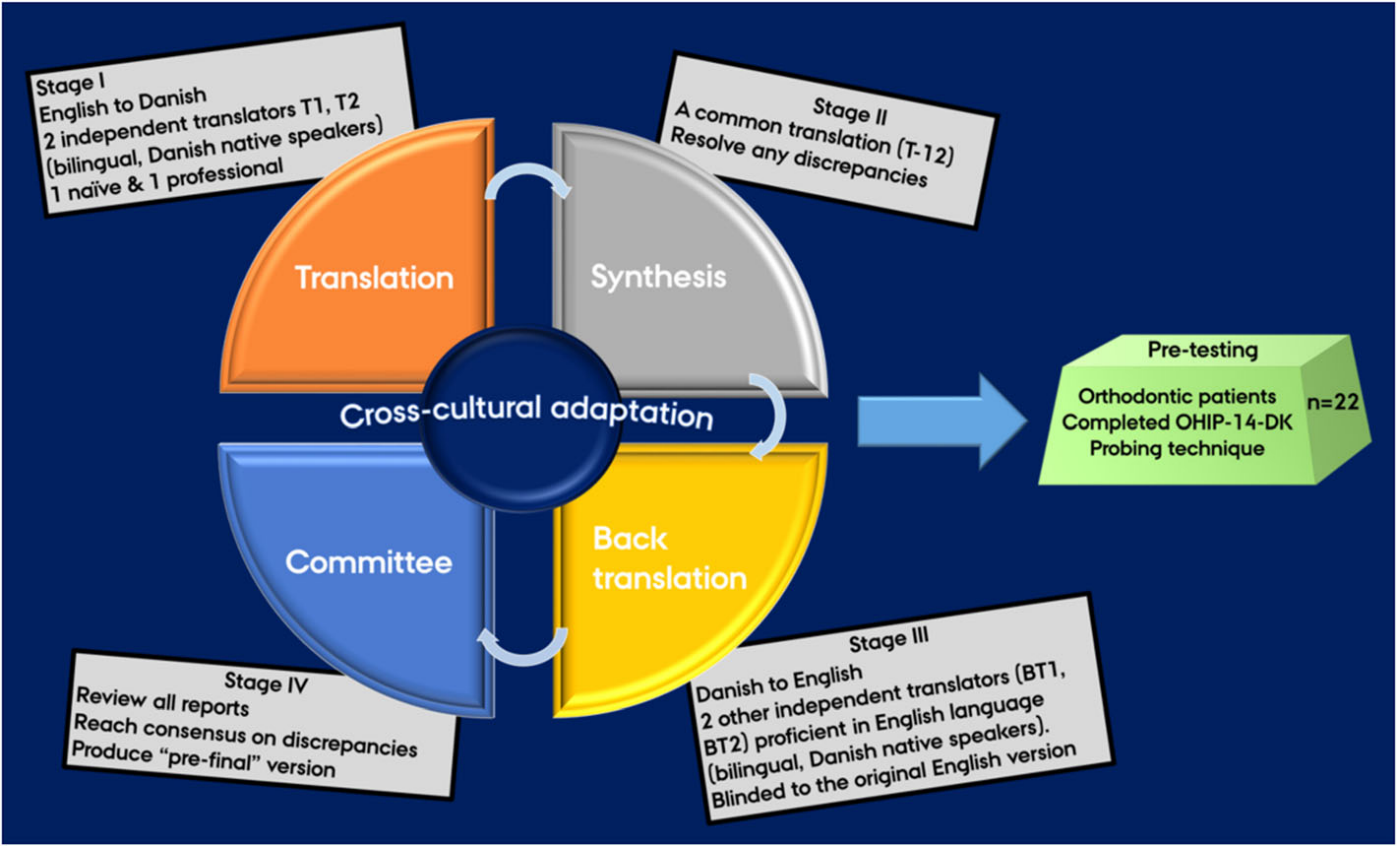
Process of translation and cross-cultural adaptation of the OHIP-14 questionnaire into Danish

Following the guidelines for the process of cross-cultural adaptation of self-reported measures proposed by Beaton et al. [15], the process of translation and cross-cultural adaptation comprised five stages. Stages I-IV involve the translation process, whereas stage V is the pre-testing phase performed to ensure quality in the translated Danish version by adaptation to the target population. Figure 1 outlines the study process.

### Stage I*:* Initial translation

An initial forward translation was performed by two bilingual native speakers, who independently translated the English questionnaire version into Danish (T1, T2). Translators were instructed to place emphasis on conceptual rather than literal equivalence, and the choice of wording and phraseology was to be simple, clear and compatible with a 12-year and above reading age. The translators were of different backgrounds: One was a language expert with no medical or clinical background (a naïve translator) and was unaware of the concepts being targeted, whereas the other was a healthcare worker who was aware of the concepts. Both were unfamiliar with the questionnaire. Each translator enclosed a written report on the translation identifying challenging phrases and uncertainties, as well as the reasons for their decisions.

### Stage II: Synthesis of the translations

Working from the original English questionnaire and the two forward translations (T1, T2), a comparison was made, and discrepancies were resolved through discussion and preparing a joint written report, yielding one common translation (T-12).

### Stage III: Back translation

To ensure a consistent translation of the original questionnaire, the common translation (T-12) was translated back into English language by two other independent translators (orthodontists), who were bilingual native speakers (proficient in English language) (BT1, BT2). Neither of them had knowledge of the original version, nor were they aware or informed of the concepts explored. Both were completely blind to the original English version. Each of them produced a written report of the translation completed.

### Stage IV: Expert committee

An expert committee consisting of two dentists and the forward and backward translators compared all translated and back-translated versions (T1, T2, T-12, BT1 and BT2), together with their corresponding written reports, with the original English version. Challenges and conceptual equivalences were resolved, yielding a pre-final version “OHIP-14-DK” ready for field testing. Among others, the committee discussed wording options that might help clarify an item and confirmed the equivalence in four areas [27]; semantic, idiomatic, experiential and conceptual equivalence. After debating the individual differences between the translations, documenting alternatives and decisions, four minor wording changes relevant to idiomatic equivalence was made. Adjustments took into account Danish language usage and conceptual equivalences aiming to avoid disagreement with the original OHIP-14. As the general recommendation for questionnaires must be understandable for a 12-year old, the pre-final version was evaluated for its readability level using an electronic readability test tool (https://www.webfx.com/tools/read-able/). Before completing stage V, ten individuals (colleagues at the Section of Orthodontics: secretaries and dental assistants) volunteered to read the pre-final Danish version and give their opinion to identify any misunderstandings or ambiguous wording that could reveal deviations in the translation.

### Stage V: Test of the pre-final version

In the final stage of the adaptation process, the translated version was tested to ensure quality in the content validity within the targeted population (conceptual equivalence with the original questionnaire). The pre-final Danish version of the OHIP-14-DK was administered to a random sample of 22 patients aged 12 years and above receiving orthodontic treatment at the Section of Orthodontics at the time of the testing of the translated Danish version. All patients were native Danes. Patients were approached at the dental chair during their regular orthodontic treatment visit by one researcher (AG); they were given a standard oral explanation of the questionnaire, agreed to participate; and then each patient self-completed the questionnaire. Responses to each item were scored on a five-point Likert scale (never to very often: 0 = never, 1 = hardly ever; 2 = occasionally; 3 = fairly often; 4 = very often). Afterwards, the same researcher immediately interviewed each patient individually (probing technique), focusing on what he/she thought was meant by each item and the chosen response to assess whether there were any difficulties in understanding any item or wording in the questionnaire. In addition, all patients completed the questionnaire again at a one-week interval.

### Scoring method and data analysis

The number of negative impacts was recorded as present for any item if the reported answers were “fairly often” or “very often” (≥ 3 on the Likert scale), while a positive impact was determined by the reported answers “never”, or “hardly ever” or “occasionally”. The percentage of patients reporting a negative impact on one or more items was calculated using the simple-count method [20]. The item response score for each item was multiplied by its relevant weight and summed to produce subscale scores. The weighted OHIP-14 total score was calculated by summing the weighted score of each subscale (weighted-standardized method). Data collection and management were performed using the Research Electronic Data Capture (REDCap) tool hosted at Aarhus University [28, 29].

Statistical analyses were performed using the Stata Statistical Software (StataCorp. 2017, version 15, College Station, TX, USA). Descriptive statistics were computed. The negative and positive impacts were described using frequency distribution. The internal consistency of the OHIP-14-DK was explored using Cronbach’s alpha. The stability in the pattern of response of the test re-test was assessed using Spearman’s correlation coefficient. Consistency of agreement was measured using intra-class correlation coefficient (ICC) with 95% confidence interval (CI) [30], computed using the “two-way mixed effects model”, with one rater (k = 1), across 22 subjects.

## Results

The translation and back-translation were very similar. Discrepancies were resolved with only minor changes being made. The ten volunteers approved the pre-final version “OHIP-14-DK” (see Appendix A.2.). Thus, no changes were necessary. The readability test (see Appendix B) showed that the pre-final version had an average level of about eight and was easily comprehended by a 13–14-year-old (8^th^ grade) individual.

All 22 patients (Table 1) self-completed the 14-item questionnaire with no missing answers, corresponding to a 100% response rate. Out of the 308 responses, 14 responses (4.5%) represented negative impacts as “fairly often” or “very often” for one or more items; 3.5% (11 responses) reported “fairly often” (score = 3) and 1% (3 responses) “very often” (score = 4).

**Table 1:** Sample characteristics: distribution of participants by number, gender and age

n=	22
**Mean age (years) ± SD**	24.7 **±** 14.8
**Range (years)**	12.9–58.4
**Gender**	
**F**	14
**M**	8

Interviews of patients confirmed that the questions were understandable without inducing reluctance or hesitation; no adjustments were needed. The pre-testing thus demonstrated that the Danish version had a good face and content validity, and was adapted to Danish culture.

### Reliability

Cronbach’s alpha and Spearman’s correlation coefficients are provided in Table 2. The values of Cronbach’s alpha fell in the 0.75–0.84 range, indicating an adequate-to-good internal consistency of the subscales when used in this setting. Spearman’s correlation coefficient was 0.77 for the total OHIP score. The ICC for the total OHIP-14-DK score was 0.91 (95% CI: 0.77–0.96).

**Table 2:** The total OHIP-14 score and its subscales with means, standard deviations (SD) and 95% confidence intervals (CI). Internal consistency (Cronbach’s alpha) and Spearman’s correlation coefficient

OHIP-14	Mean ± SD	95% CI	Internal consistency (Cronbach’s alpha)	Spearman’s correlation coefficient
***Total score***	5.54 **±** 3.70	3.90–7.20	0.82	0.77
Subscales				
***Functional limitation***	0.28 **±** 0.40	0.10–0.46	0.84	0.82
***Physical pain***	1.40 **±** 0.93	0.99–1.82	0.80	0.62
***Psychological discomfort***	1.18 **±** 0.95	0.76–1.60	0.75	0.83
***Physical disability***	0.70 **±** 0.69	0.39–1.01	0.83	0.74
***Psychological disability***	0.91 **±** 0.83	0.54–1.28	0.77	0.80
***Social disability***	0.63 **±** 0.72	0.31–0.95	0.77	0.62
***Handicap***	0.44 **±** 0.60	0.17–0.71	0.80	0.81

## Discussion

There is a great demand for cross-culture QoL measures. This study handles the translation and cross-cultural adaptation of a previously validated OHIP-14 (English) into the language and cultural context of a Danish population. The guideline recommended by Beaton et al. was followed to adapt the OHIP-14 cross-culturally [15]. We did not encounter notable difficulties in this translation and adaptation process.

OHRQoL instruments are multidimensional [31]. Dimensions of OHRQoL are an informative way of profiling important domains of oral health. The OHIP authors grouped the questionnaire items into seven domains based on expert opinion and a conceptual model of oral health [18]. Subsequent qualitative studies have provided evidence for the multidimensional nature of the OHIP scores, but their findings did not agree on the number of dimensions. On the one hand, studies of OHIP-14 responses by UK and Australian general population subjects led authors to accept the original seven-dimensional structure [32]. On the other hand, experts found that only four dimensions were needed when assigning OHIP items to each of the original seven domains [33]. However, some investigators used the original seven-dimension framework of the OHIP-14, while others believe that the shortened form does not contain sufficient indicators and is therefore unable to identify an *orofacial appearance* dimension which deserves a place in the theoretical structure [34]. John et al. [34] explored the dimensional structure of the OHIP and concluded that the use of the OHIP-14 as a single OHIP summary score is sufficient to characterize OHRQoL.

The OHIP weights reflect the relative severity of the items. However, using the unweighted scores (simple scoring method) is considered by others to be a more straightforward method [35, 36]. Indeed, calculation of weights and score responses can be cumbersome and time consuming if handled manually. In the present study, the questionnaire was digitized, and all data were entered and manipulated through RedCap, which made the calculation feasible, automatic and less time consuming. Therefore, we used the original method of calculating OHIP scores [20]. For the second evaluation, all patients received the questionnaire by personal email. In the present study, we did not consider adapting the weights of scores to the cultural context; we applied the original weights. We do not believe this would have affected the instrument as the final version did not differ much from the original one; items were the same, and no additional wording or items were introduced.

In a newly translated questionnaire version, the comprehensibility is of major importance. Colleagues who were not undergoing orthodontic treatment were considered a useful first group for additional face validity testing before pre-testing [37]. Afterwards, orthodontic patients were involved in the pre-testing as they can judge the comprehensibility, relevance and completeness, especially because they represent the typical target group of such a questionnaire. Ideally, between 30 and 40 subjects should be tested [15], which is consistent with the number reached, combining the first step (10 subjects) and the pre-testing (22 subjects). Other authors believe that 15–30 subjects are sufficient [38]. Thus, our sample size was appropriate for pre-testing.

Spearman’s correlation coefficients showed a strong correlation, indicating that the OHIP-14-DK is reproducible on different occasions. Furthermore, the ICC estimate for a single measurement indicated “excellent” reliability.

The OHIP-14 has been used in many fields of dentistry, but it is not so common within orthodontics. Thus, only one recent study has assessed the perception of patients wearing vacuum-formed retainers using this tool [39], whereas other studies have reported its use in orofacial pain patients [40, 41], prosthodontic patients [42] and periodontal patients [43]. A systematic review and meta-analysis [44] concluded that OHIP-14 scores were significantly lower after patients had received treatment for malocclusion and that individuals without a malocclusion/orthodontic treatment need had scored lower than those with such a condition. In the present study, our selected orthodontic population had a range of malocclusions and were undergoing treatment. They showed a mean OHIP-14 total score of 5.5 points, which supports the fact that malocclusion has a significant impact on patients’ emotional and social well-being [45–48]. This might imply that orthodontic patients have a hard time adapting their social life with their brackets or orthodontic treatment in general. Furthermore, OHRQoL research is needed in the orthodontic field.

In a literature review, Guillemin et al. [27] stated that a standardized approach to cross-cultural adaptation of OHRQoL instruments does not exist. The authors proposed a guideline comprised by five steps for translation and cross-cultural adaptation of OHRQoL measures. Prior studies that culturally adapted a measure used different methodologies. In addition, authors often did not give the readers essential information to comprehend the strength of the translation. However, it remains unclear which elements are essential and which are merely supplementary for this process: whether reliability, validity and sensitivity should also be considered in the cross-cultural adaptation process is a matter of controversy. The aspects of validity considered in this pilot study were face and content validity; this approach was adopted to ascertain the appropriateness and relevance of the content, ensuring feasibility, readability and clarity of language to the participating audience. However, the translation process is the first step in the three-step process adopted by the International Society for Quality of Life Assessment (IQOLA) project [49]. Hence, OHIP-14-DK needs further testing as it lacks validation of its psychometric properties.

## Conclusions

OHIP-14-DK was adapted to fit Danish culture, proved to be face and content valid, and showed good internal consistency and excellent reliability. However, its psychometric properties still need to be tested.

## Supplementary information

**Additional file 1: Appendix A.** OHIP-14 questionnaires; the original English version and the translated Danish version (OHIP-14-DK).

**Additional file 2: Appendix B.** Readability test results.

## Data Availability

The datasets used and/or analysed during the current study are available from the corresponding author on reasonable request.

## Abbreviations

QoL: Quality of life
WHO: World Health Organization
HRQoL: Health-related quality of life
OHRQoL: Oral health-related quality of life
OHIP-49: Oral Health Impact Profile-49
OHIP-14: Oral Health Impact Profile-14
OHIP-14-DK: Oral Health Impact Profile-14-Danish version
IQOLA: International Society for Quality of Life Assessment
