# Supplementary material for: A Danish version of the oral health impact profile-14 (OHIP-14): translation and cross-cultural adaptation
Source: BMC Oral Health. 2020 Sep 10;20:254. doi: 10.1186/s12903-020-01242-z (PMC7488136; doi:10.1186/s12903-020-01242-z)
Supplement: Supplementary file 1 — Additional file 1: Appendix A. OHIP-14 questionnaires; the original English version and the translated Danish version (OHIP-14-DK). [file 12903_2020_1242_MOESM1_ESM.pdf]

Appendix A- OHIP-14 questionnaires; the original English version and the translated Danish version (OHIP-14-DK).

1. **OHIP-14 questionnaire-English version (Slade et al. 1997)**

During the last 12 months, how often has the following occurred?		
**Dimension**	**Question**	**Weight**
Functional limitation	Have you had trouble *pronouncing any words* because of problems with your teeth, mouth or dentures?	0.51
	Have you felt that your *sense of taste* has worsened because of problems with your teeth, mouth or dentures?	0.49
Physical pain	Have you had *painful aching* in your mouth?	0.34
	Have you found it *uncomfortable to eat any foods* because of problems with your teeth, mouth or dentures?	0.66
Psychological discomfort	Have you been *self-conscious* of your teeth, mouth or dentures?	0.45
	Have you *felt tense* because of problems with your teeth, mouth or dentures?	0.55
Physical disability	Has your *diet been unsatisfactory* because of problems with your teeth, mouth or dentures?	0.52
	Have you had to *interrupt meals* because of problems with your teeth, mouth or dentures?	0.48
Psychological disability	Have you found it *difficult to relax* because of problems with your teeth, mouth or dentures?	0.60
	Have you been a bit *embarrassed* because of problems with your teeth, mouth or dentures?	0.40
Social disability	Have you been a bit *irritable with other people* because of problems with your teeth, mouth or dentures?	0.62
	Have you had *difficulty doing your usual jobs* because of problems with your teeth, mouth or dentures?	0.38
Handicap	Have you felt that life in general was *less satisfying* because of problems with your teeth, mouth or dentures?	0.59
	Have you been *totally unable to function* because of problems with your teeth, mouth or dentures?	0.41
**Responses are made on a 5-point scale, coded 0=never, 1=hardly ever, 2=occasionally, 3=fairly often, 4=very often.**		

2. **OHIP-14 questionnaire-Danish version (OHIP-14-DK).**

Dette spørgeskema handler om sammenhæng mellem livskvalitet og sundhed i mundhulen (Oral Health Related Quality of Life (OHRQoL)). Besvar venligst spørgsmålene neden for om din orale sundhed med udgangspunkt i <u>det seneste år</u>. Hvert spørgsmål skal besvares ved at angive en enkelt svarmulighed.

	**Spørgsmål**	**Svar**
**1**	Har du haft problemer med at *udtale ord* på grund af problemer med dine tænder, mund eller gebis?	☐ 0 = Aldrig<br>☐ 1 = Næsten aldrig<br>☐ 2 = Nogle gange<br>☐ 3 = Temmelig ofte<br>☐ 4 = Meget ofte
**2**	Har du følt, at din *smagsevne* er forringet på grund af problemer med dine tænder, mund eller gebis?	☐ 0 = Aldrig<br>☐ 1 = Næsten aldrig<br>☐ 2 = Nogle gange<br>☐ 3 = Temmelig ofte<br>☐ 4 = Meget ofte
**3**	Har du haft stærke *smerter* i din mund?	☐ 0 = Aldrig<br>☐ 1 = Næsten aldrig<br>☐ 2 = Nogle gange<br>☐ 3 = Temmelig ofte<br>☐ 4 = Meget ofte
**4**	Har det været *ubehageligt at spise mad p*å grund af problemer med dine tænder, mund eller gebis?	☐ 0 = Aldrig<br>☐ 1 = Næsten aldrig<br>☐ 2 = Nogle gange<br>☐ 3 = Temmelig ofte<br>☐ 4 = Meget ofte
**5**	Har du følt dig *forlegen* på grund af dine tænder, mund eller gebis?	☐ 0 = Aldrig<br>☐ 1 = Næsten aldrig<br>☐ 2 = Nogle gange<br>☐ 3 = Temmelig ofte<br>☐ 4 = Meget ofte
**6**	Har du følt dig *anspændt* på grund af problemer med dine tænder, mund eller gebis?	☐ 0 = Aldrig<br>☐ 1 = Næsten aldrig<br>☐ 2 = Nogle gange<br>☐ 3 = Temmelig ofte<br>☐ 4 = Meget ofte
**7**	Har din *kost været utilfredsstillende* på grund af problemer med dine tænder, mund eller gebis?	☐ 0 = Aldrig<br>☐ 1 = Næsten aldrig<br>☐ 2 = Nogle gange<br>☐ 3 = Temmelig ofte<br>☐ 4 = Meget ofte
**8**	Har du måtte *afbryde måltider* på grund af problemer med dine tænder, mund eller gebis?	☐ 0 = Aldrig<br>☐ 1 = Næsten aldrig

		☐ 2 = Nogle gange<br>☐ 3 = Temmelig ofte<br>☐ 4 = Meget ofte
**9**	Har du *haft svært ved at slappe af* på grund af problemer med dine tænder, mund eller gebis?	☐ 0 = Aldrig<br>☐ 1 = Næsten aldrig<br>☐ 2 = Nogle gange<br>☐ 3 = Temmelig ofte<br>☐ 4 = Meget ofte
**10**	Har du følt dig lidt *flov* på grund af problemer med dine tænder, mund eller gebis?	☐ 0 = Aldrig<br>☐ 1 = Næsten aldrig<br>☐ 2 = Nogle gange<br>☐ 3 = Temmelig ofte<br>☐ 4 = Meget ofte
**11**	Har du været lidt *irritabel over for andre personer* på grund af problemer med dine tænder, mund eller gebis?	☐ 0 = Aldrig<br>☐ 1 = Næsten aldrig<br>☐ 2 = Nogle gange<br>☐ 3 = Temmelig ofte<br>☐ 4 = Meget ofte
**12**	Har du haft *svært ved at løse dine normale opgaver* på grund af problemer med dine tænder, mund eller gebis?	☐ 0 = Aldrig<br>☐ 1 = Næsten aldrig<br>☐ 2 = Nogle gange<br>☐ 3 = Temmelig ofte<br>☐ 4 = Meget ofte
**13**	Har du følt at livet generelt har været *mindre tilfredsstillende* på grund af problemer med dine tænder, mund eller gebis?	☐ 0 = Aldrig<br>☐ 1 = Næsten aldrig<br>☐ 2 = Nogle gange<br>☐ 3 = Temmelig ofte<br>☐ 4 = Meget ofte
**14**	Har du været *helt ude af stand til at fungere* på grund af problemer med dine tænder, mund eller gebis?	☐ 0 = Aldrig<br>☐ 1 = Næsten aldrig<br>☐ 2 = Nogle gange<br>☐ 3 = Temmelig ofte<br>☐ 4 = Meget ofte
