# Supplementary figures and images for: A Danish version of the oral health impact profile-14 (OHIP-14): translation and cross-cultural adaptation
Source: BMC Oral Health. 2020 Sep 10;20:254. doi: 10.1186/s12903-020-01242-z (PMC7488136; doi:10.1186/s12903-020-01242-z)

## Appendix B

Readability test results <https://www.webfx.com/tools/read-able/>

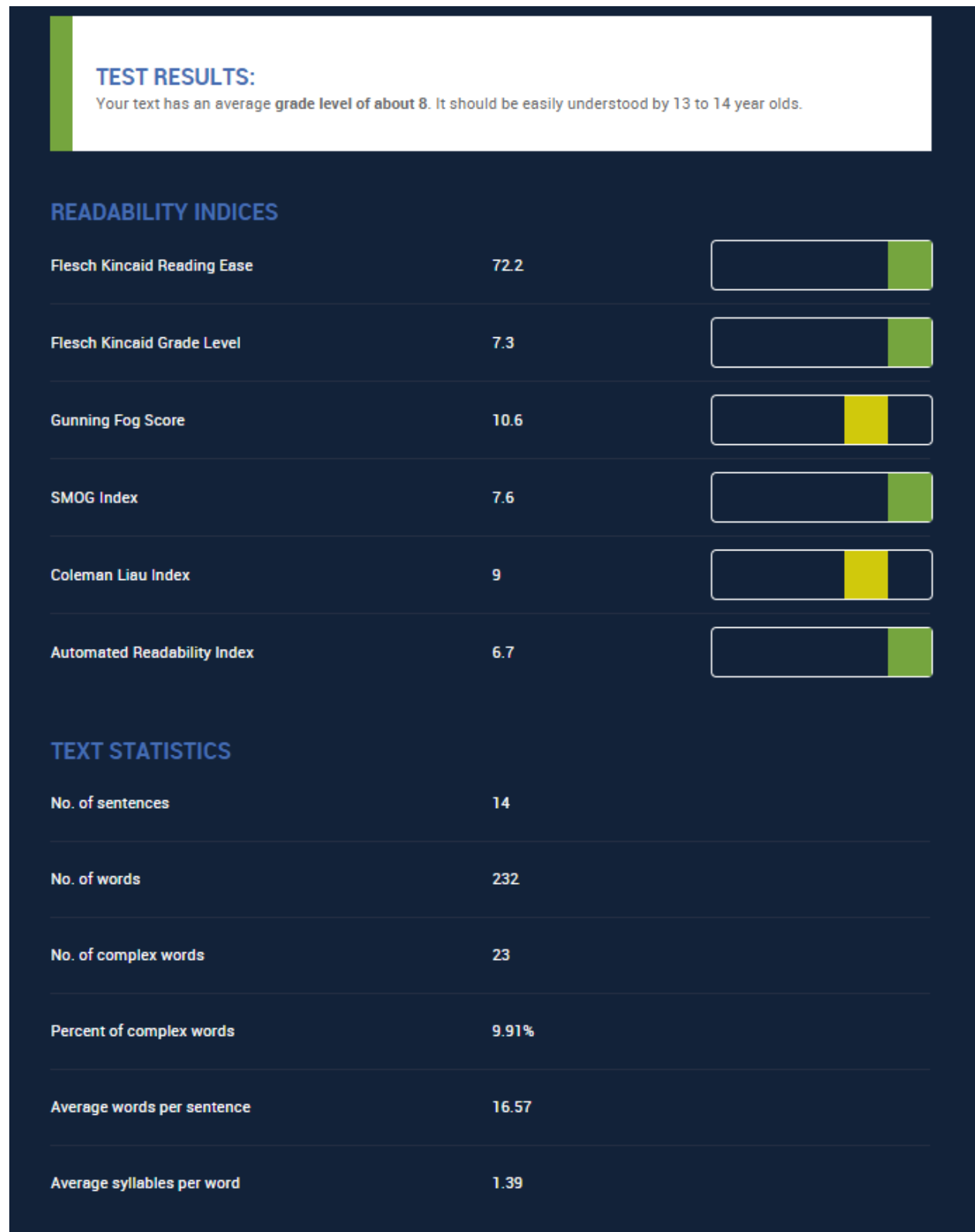

Supplement: Supplementary file 2 — Additional file 2: Appendix B. Readability test results. [file 12903_2020_1242_MOESM2_ESM.pdf]
